# Association Between Cigarette Smoking and Carotid Plaque Stiffness Assessed Using Ultrasound Shear-Wave Elastography

**DOI:** 10.3390/tomography12090135

**Published:** 2026-09-16

**Authors:** Salahaden R. Sultan, Adel Alzahrani

**Affiliations:** 1 Department of Radiologic Sciences, Faculty of Applied Medical Sciences, King Abdulaziz University, Jeddah 21589, Saudi Arabia; 2 Department of Radiology, King Abdulaziz University Hospital, King Abdulaziz University, Jeddah 21589, Saudi Arabia; 3 Department of Diagnostic Radiology and Imaging, King Abdullah Medical City, Makkah 21955, Saudi Arabia; alzahrani.a7@kamc.med.sa

**Keywords:** carotid plaque, smoking, shear-wave elastography, ultrasound

## Abstract

Background: Cigarette smoking alters carotid plaque composition. Two-dimensional ultrasound shear-wave elastography (2D-SWE) is a promising non-invasive imaging technique for quantifying plaque stiffness. This prospective study aimed to determine whether carotid plaque stiffness assessed using 2D-SWE is associated with smoking status. Methods: A total of 132 carotid plaques were evaluated from 93 patients who underwent carotid ultrasound 2D-SWE. Demographic and clinical data were collected, and patients were classified as current cigarette smokers (n = 45) and non-smokers (n = 48). A total of 65 carotid plaques in smokers and 67 in non-smokers were evaluated. Plaque stiffness was quantified using shear-wave velocity (m/s), with three SWE measurements obtained from each plaque. Demographic and clinical characteristics and plaque stiffness were compared according to smoking status, and correlations between smoking status and plaque stiffness were evaluated. Results: A total of 396 SWE measurements were obtained from 132 carotid plaques (195 in cigarette smokers and 201 in non-smokers). Plaques in smokers demonstrated significantly greater stiffness than those in non-smokers, as indicated by shear-wave velocity (smokers: median 4.17 m/s, interquartile range (IQR) 1.94 vs. non-smokers: median 2.91 m/s, IQR 1.81, Z = −5.36, *p* < 0.001). Smoking was positively correlated with shear-wave velocity (r = 0.27, *p* < 0.001). No significant differences were observed between smokers and non-smokers in age, sex, hypertension, or diabetes mellitus. Conclusions: Cigarette smoking was associated with increased carotid plaque stiffness measured using ultrasound 2D-SWE. These findings suggest that SWE may provide a quantitative imaging marker of smoking-related differences in carotid plaque composition. Larger studies incorporating smoking duration and cumulative exposure are warranted to determine their clinical significance.

## Simple Summary

Carotid plaques are build-ups that develop within the neck arteries supplying blood to the brain and increase the risk of stroke. Cigarette smoking may alter their composition and mechanical properties. Conventional ultrasound mainly assesses plaque appearance, whereas ultrasound shear-wave elastography (SWE) provides an objective measurement of plaque stiffness. In this study, we used this technique to assess 132 carotid plaques in 93 patients, including 45 current smokers and 48 non-smokers. Plaques in current smokers were significantly stiffer than those in non-smokers. Our findings suggest that adding SWE to routine carotid ultrasound may help identify smoking-related differences in plaque tissue. However, greater stiffness does not directly indicate whether a plaque is stable or vulnerable. Larger studies assessing smoking alongside other cardiovascular risk factors are needed to determine how SWE-measured stiffness relates to plaque composition and vulnerability.

## 1. Introduction

Atherosclerosis is a leading cause of morbidity and mortality worldwide, with cigarette smoking representing a major preventable risk factor for coronary artery disease, ischemic stroke, and peripheral arterial disease [1]. Cigarette smoking contributes to atherosclerosis through multiple interacting mechanisms, including sympathetic activation, endothelial dysfunction, oxidative stress, vascular inflammation, lipid oxidation, platelet activation, and impaired fibrinolysis [2,3,4], and may modify the composition and mechanical properties of atherosclerotic plaques [5,6,7]. It has been reported that smoking promotes the progression of carotid plaque and alters its composition [8,9].

B-mode ultrasound enables the evaluation of carotid plaque size, echogenicity, and surface characteristics [10]. A study assessed the association between cigarette smoking and carotid plaque morphology on ultrasound and reported that current smokers were more likely to exhibit either echolucent or highly echogenic plaques, whereas former smoking was predominantly associated with more echogenic plaques [11]. These appearances on ultrasound imaging may reflect differences in plaque composition, including lipid accumulation, fibrosis, and calcification [12]. Ultrasound shear-wave elastography (SWE) extends conventional B-mode ultrasound by providing a quantitative assessment of carotid plaque mechanical properties that cannot be inferred directly from plaque morphology or echogenicity [13]. SWE uses acoustic radiation force to generate shear waves whose propagation velocity increases with tissue stiffness, enabling localized quantification of plaque stiffness as shear-wave velocity (m/s) or estimated Young’s modulus (kPa) reflecting the integrated stiffness of an arterial segment [14]. Recent ultrasound elastography studies have demonstrated greater carotid and aortic wall stiffness in smokers than in non-smokers [15,16] and that SWE-derived carotid wall elasticity modulus has been associated with smoking status, with smokers exhibiting significantly higher values than non-smokers [17]. However, these studies focused primarily on diffuse arterial wall properties rather than the local mechanical characteristics of focal carotid plaques; consequently, the relationship between cigarette smoking and carotid plaque stiffness remains unclear. Therefore, this study evaluated the association between cigarette smoking and carotid plaque stiffness assessed by ultrasound 2D-SWE.

## 2. Methods

**Figure 1:**
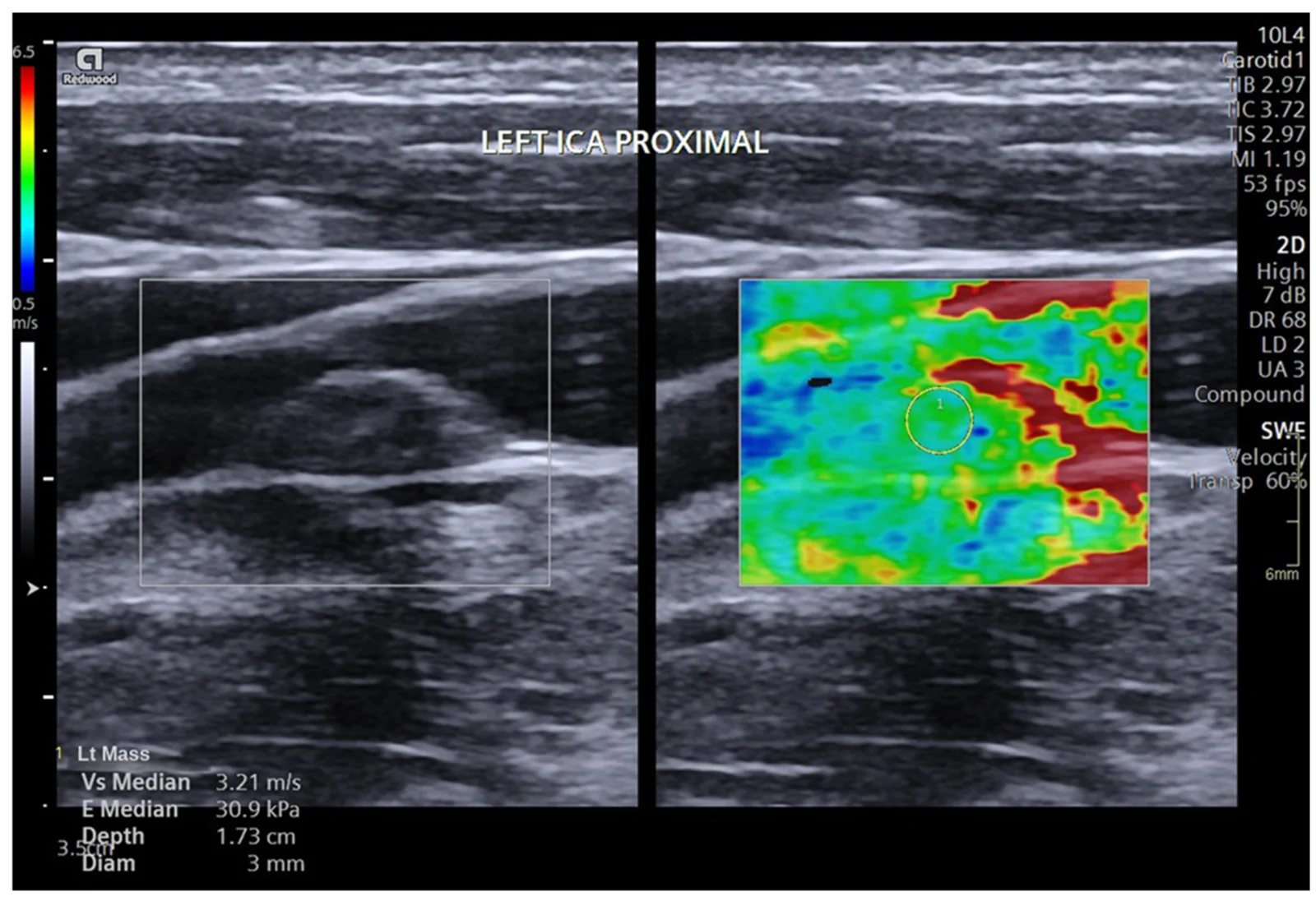
B-mode and shear-wave elastography (SWE) images of a plaque in the proximal left internal carotid artery of a non-smoker. **Left panel**: B-mode image showing the carotid plaque within the square SWE acquisition box. **Right panel**: Corresponding SWE color map overlaid on the B-mode image, with the circular 3 mm sampling ROI positioned within the plaque.

**Figure 2:**
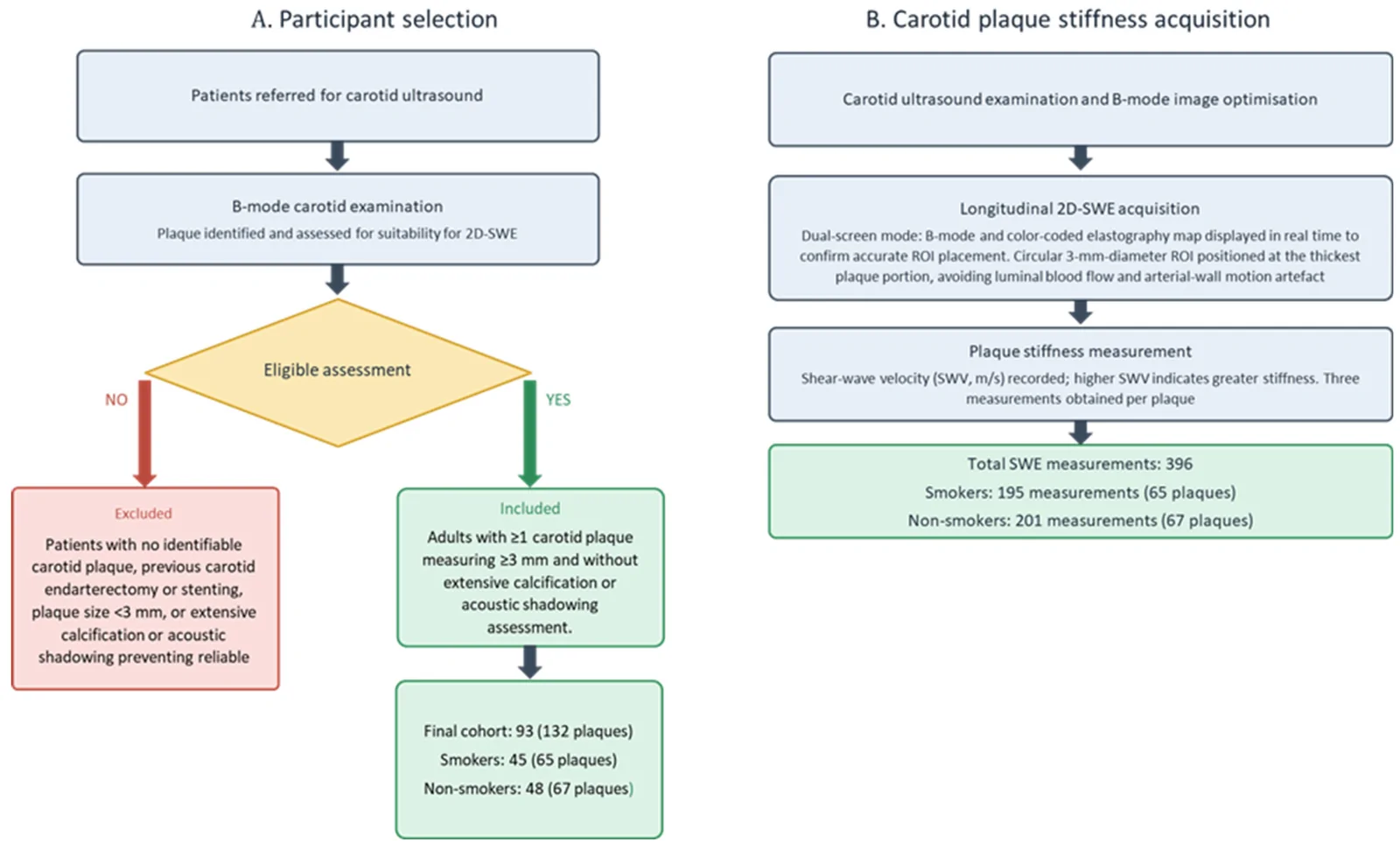
Schematic illustration of the study methodology, including participant selection (**A**) and carotid plaque stiffness acquisition (**B**).

This prospective observational pilot study was conducted in accordance with the Declaration of Helsinki and the International Council for Harmonization Good Clinical Practice guidelines. Ethical approval was obtained from the Institute Review Board of Research Ethics Committee at King Abdullah Medical City (IRB No 23-1177). Adult patients referred to the ultrasound department for carotid examination were recruited after providing written informed consent. Patients were eligible if they had at least one carotid atherosclerotic plaque identifiable on B-mode ultrasound and suitable for assessment using ultrasound 2D-SWE. Exclusion criteria were the absence of an identifiable carotid plaque, previous carotid endarterectomy or stent placement, extensive calcification or acoustic shadowing that prevented reliable plaque assessment, or a plaque too small to accommodate the minimum 3 mm region of interest (ROI). Demographic and clinical information, including age, gender, body mass index (BMI), smoking status, hypertension, and diabetes mellitus, were collected. Participants were classified according to their smoking status as current active cigarette smokers or non-smokers. All bilateral carotid ultrasound examinations and SWE measurements were performed by a single consultant sonographer with 12 years of experience in vascular ultrasound and specific training and experience in ultrasound SWE. Participants were examined in the supine position with their necks slightly extended and arms resting comfortably at their sides. Sufficient coupling gel and minimal transducer pressure were used to maintain adequate contact while avoiding plaque compression and related measurement artifacts. The common, internal, and external carotid arteries were examined using a high-resolution ACUSON Redwood ultrasound system (Siemens Healthineers, Erlangen, Germany) equipped with a 10^−4^ MHz linear-array transducer. Imaging parameters, including depth, focal position, overall gain, time-gain compensation, and dynamic range, were adjusted to optimize B-mode image quality. Carotid plaque stiffness was assessed using 2D-SWE in the longitudinal plane. Dual-screen mode was activated for all acquisitions, displaying the conventional B-mode grayscale image alongside the co-registered, color-coded elastography map in real time, which allowed the operator to visually confirm accurate ROI placement and sampling within the plaque. A circular ROI was placed within the plaque for stiffness sampling; the ROI diameter was fixed at 3 mm, the minimum size permitted by the manufacturer’s software, and was positioned at the thickest portion of the plaque to maximize tissue sampling within the ROI while minimizing motion artifact from adjacent luminal blood flow and the arterial wall. Plaque stiffness was quantified using shear-wave velocity (SWV; m/s), with higher values indicating greater stiffness (Figure 1). This is consistent with the acquisition protocol established in our previous study in which reproducibility was excellent [18]. Three consecutive technically adequate measurements were obtained from each plaque. In total, 396 SWE measurements were obtained from 132 carotid plaques. Image interpretation and quantitative analysis were performed blinded to participants’ clinical characteristics and smoking status. Figure 2 presents a schematic overview of the study methodology, including participant selection, exclusion criteria, and the principal steps involved in carotid plaque 2D-SWE acquisition.

### Statistical Analysis

Data normality was evaluated using the Shapiro–Wilk test. Continuous variables with a normal distribution are presented as mean ± standard deviation, while non-normally distributed variables are expressed as median (interquartile range). Demographic and clinical characteristics were compared between smokers and non-smokers using the independent-samples *t*-test for continuous variables and the chi-square test for categorical variables. All three measurements obtained from each plaque were included in the final statistical analysis. Comparisons of carotid plaque stiffness, measured as shear-wave velocity, were performed using the Mann–Whitney *U* test. The association between smoking status and shear-wave velocity was examined using the point-biserial correlation coefficient. Within-plaque reproducibility of the triplicate readings was assessed using the intraclass correlation coefficient (ICC). Statistical analyses were conducted using IBM SPSS Statistics version 21.0 and GraphPad Prism version 7.

## 3. Results

### 3.1. Overall Patient and Carotid Plaque Characteristics

The study included 93 patients with 132 carotid plaques. The mean (±standard deviation, SD) age was 68.4 ± 10.1 years (range, 39–96 years). Of the patients, 73 (78.5%) were men and 20 (21.5%) were women. Men contributed 108 plaques (81.8%), whereas women contributed 24 plaques (18.2%). Hypertension was documented in 70 patients (75.3%), while 62 (66.7%) had diabetes mellitus. A total of 45 patients (48.4%) were current active smokers and 48 (51.6%) were non-smokers. The mean shear-wave velocity was 3.87 ± 1.40 m/s (95% CI, 3.73–4.01 m/s), with a median of 3.79 m/s (IQR, 2.57 m/s) and a range of 1.16–6.46 m/s. Summary of patient characteristics, plaque distribution, and stiffness is presented in Table 1.

### 3.2. Patient Characteristics and Carotid Plaque Stiffness According to Smoking Status

**Figure 3:**
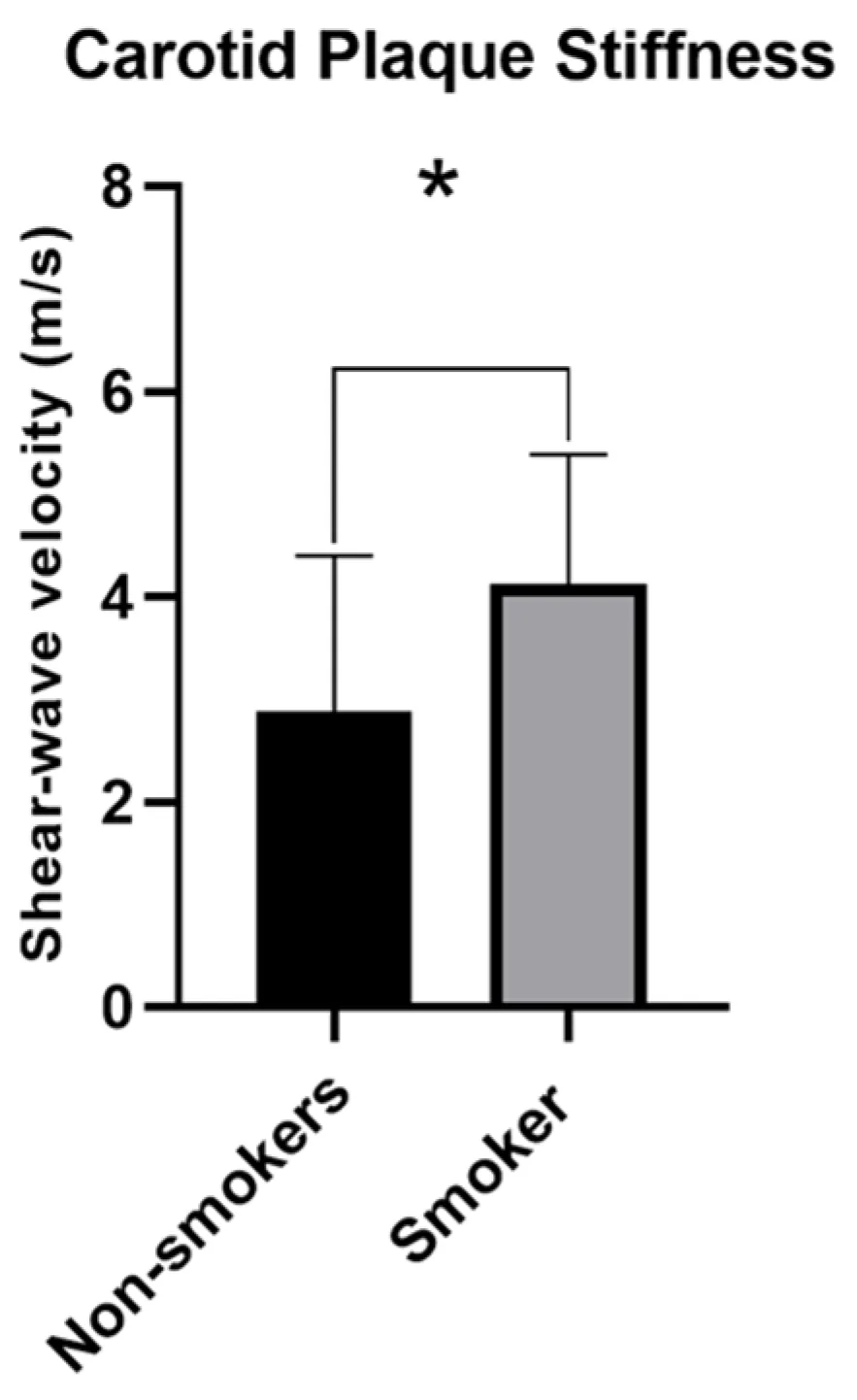
Carotid plaque stiffness measured using ultrasound shear-wave velocity and presented as the median with interquartile range. * *p* < 0.05 indicates a significant difference between smokers and non-smokers using the Mann–Whitney U test.

**Figure 4:**
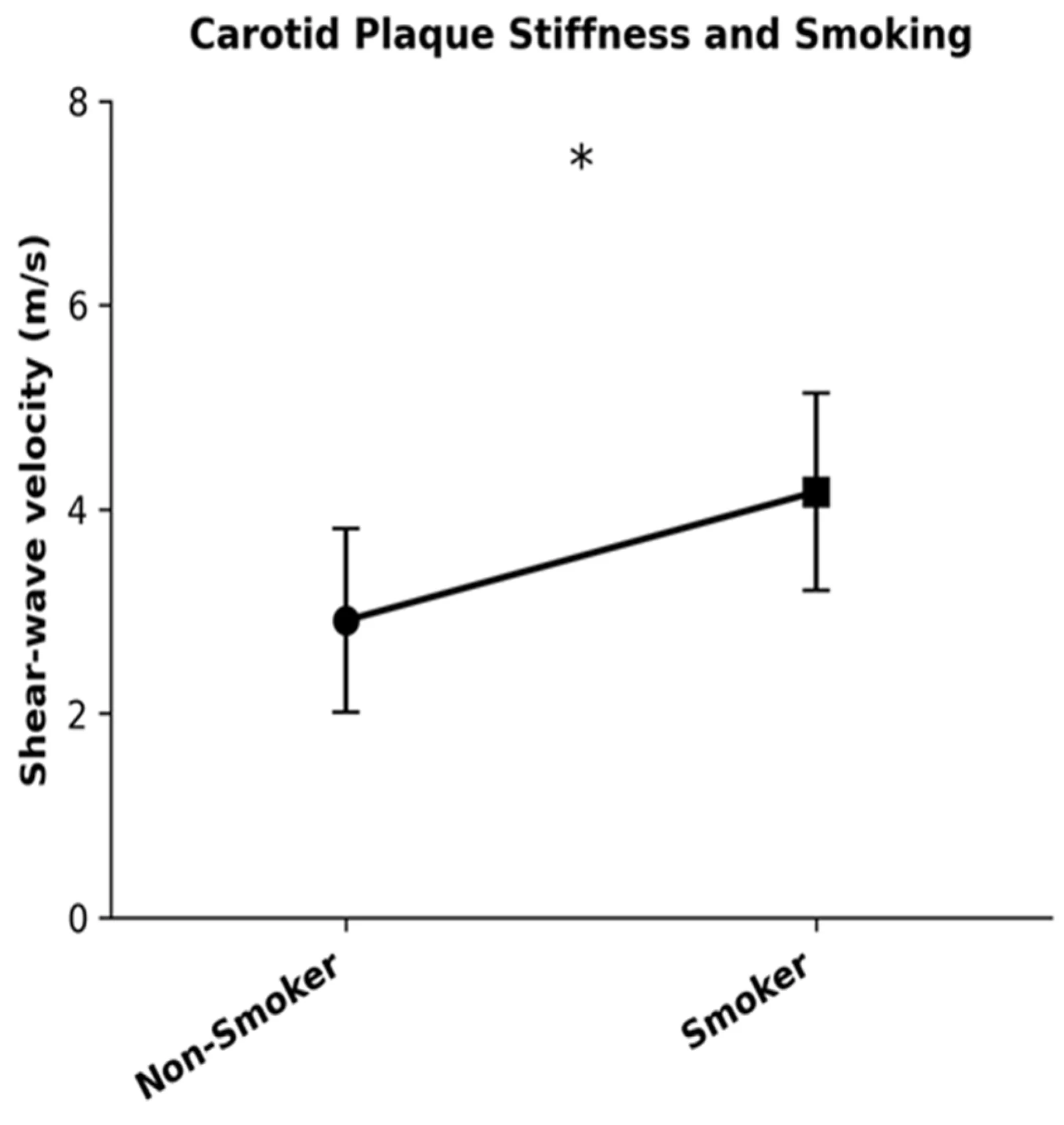
Correlation between smoking status and carotid plaque stiffness measured using shear-wave velocity. * *p* < 0.05 indicates a significant positive correlation, reflecting greater plaque stiffness among smokers. Error bars represent the interquartile range.

The study included 45 smokers and 48 non-smokers, who contributed 65 and 67 carotid plaques, respectively. The groups were comparable in age, gender distribution and prevalence of hypertension and diabetes mellitus (all *p* > 0.05). Among smokers, 36 (80.0%) were men, 35 (77.8%) had hypertension, and 33 (73.3%) had diabetes mellitus. Among non-smokers, 37 (77.1%) were men, 35 (72.9%) had hypertension, and 29 (60.4%) had diabetes mellitus. Multiple plaques were present in 12 smokers (26.7%) and 13 non-smokers (27.1%). Carotid plaques in smokers were significantly stiffer than those in non-smokers. Median shear-wave velocity was 4.17 m/s in smokers and 2.91 m/s in non-smokers, with corresponding IQRs of 1.94 and 1.81 m/s (Z = −5.36, *p* < 0.001) (Figure 3). Correlation analysis also demonstrated significant positive correlations between smoking status and shear-wave velocity (r = 0.27, 95% confidence interval 0.17 to 0.36, *p* < 0.001, Figure 4). The triplicate SWE readings demonstrated excellent within-plaque repeatability, with ICC of 0.996 (95% CI: 0.994–0.997, *p* < 0.001). Demographic and clinical characteristics, plaque distribution, and stiffness measurements stratified by smoking status are presented in Table 2.

## 4. Discussion

To our knowledge, this is the first study to specifically address the relationship between cigarette smoking and the local mechanical stiffness of carotid plaques. Plaques in cigarette smokers were significantly stiffer than those in non-smokers, with a markedly higher median shear-wave velocity. Smoking status was positively associated with carotid stiffness, and this association persisted despite similar age, BMI, sex, hypertension, and diabetes distributions in smokers and non-smokers; however, confounding by systemic inflammation and metabolic syndrome can still influence carotid plaque development and vulnerability [19,20]. These findings are consistent with, and extend, prior elastographic studies that examined diffuse carotid wall stiffness and elasticity in smokers compared with non-smokers [16,17]. These suggest that smoking-related vascular stiffening assessed using ultrasound SWE may extend from plaque-free arterial wall segments to atherosclerotic plaques, demonstrating a measurable effect of smoking on the mechanical properties of both diseased and non-diseased regions of the carotid artery.

Several interrelated biological mechanisms may explain the higher plaque shear-wave velocity observed in smokers. Recurrent endothelial injury represents an early event in smoking-related vascular remodeling, as cigarette smoking increases adhesion-molecule expression, endothelial activation, and mitochondrial dysfunction while impairing vasodilator signaling [3]. Smoking also reduces nitric oxide bioavailability by increasing reactive oxygen species and superoxide production, promoting peroxynitrite formation, depleting tetrahydrobiopterin, and inducing endothelial nitric oxide synthase uncoupling, thereby impairing endothelium-dependent vasodilation and favoring vasoconstrictor pathways [21,22,23]. This endothelial dysfunction is amplified by chronic oxidative stress and inflammation through NF-κB, MAPK, and damage-associated molecular-pattern pathways, which sustain cytokine production, leukocyte recruitment, monocyte–macrophage infiltration, NADPH oxidase activity, and superoxide generation [2,3,21]. These redox-inflammatory conditions promote vascular smooth-muscle-cell phenotypic switching, migration, autophagy, and necroptosis, with a corresponding loss of the contractile phenotype [24,25]. Subsequent extracellular matrix remodeling involves altered matrix synthesis and degradation, increased matrix-remodeling gene expression, and matrix metalloproteinase-mediated disruption of vascular structure [3,26,27,28]. Oxidative stress and inflammation promote fibrosis, collagen I and fibronectin deposition, and LOXL2-mediated cross-linking, while elastin degradation and imbalance in the MMP–TIMP–lysyl oxidase system reduce the elastin-to-collagen ratio and increase arterial stiffness [29,30]. Smoking also promotes vascular calcification, with nicotine directly inducing smooth-muscle-cell calcification through calcium-dependent NOX5 signaling, oxidative stress, extracellular-vesicle release, and osteogenic gene expression [7]. Collectively, endothelial dysfunction, nitric oxide depletion, oxidative inflammation, smooth-muscle-cell activation, extracellular-matrix remodeling, fibrosis, and calcification provide a biological explanation for the mechanically stiffer plaque environment and higher shear-wave velocity observed in smokers.

The higher plaque stiffness observed in smokers should be interpreted cautiously because arterial wall stiffness and atherosclerotic plaque stiffness, although related, are not interchangeable, and the relationship between plaque stiffness and vulnerability is not necessarily linear. Histological evidence indicates that lipid-rich, hemorrhagic, and otherwise unstable plaque regions tend to be softer than collagen-rich or calcified regions; accordingly, a lower SWE has been reported in histologically unstable carotid plaques than in stable plaques [31,32,33]. Similarly, a recent systematic review and meta-analysis found lower SWE-derived stiffness in symptomatic than in asymptomatic carotid plaques, consistent with a lipid-rich, necrotic, and rupture-prone phenotype [13]. Smoking itself has been associated with both echolucent and highly echogenic carotid plaques, suggesting a nonlinear relationship between tobacco exposure and plaque composition [11]. Thus, the higher shear-wave velocity observed in the present study may reflect a greater contribution from fibrosis or calcification and distribution of plaque components rather than plaque vulnerability; therefore, these findings should not be interpreted as indicating that plaques in smokers were necessarily either stable or vulnerable.

Furthermore, the association between smoking and greater plaque stiffness was observed in the present cohort with comparable age and BMI and similar distributions of sex, hypertension, and diabetes mellitus between smokers and non-smokers. However, age, sex, obesity, hypertension, diabetes, dyslipidemia, and metabolic syndrome may influence carotid plaque development, composition, and mechanical properties. Advancing age is associated with progressive arterial fibrosis, elastin fragmentation, and calcification, which may stiffen the vessel wall and overlying plaque [34,35]. Sex-related differences have also been described, with premenopausal women generally exhibiting more lipid-poor, mechanically favorable plaque phenotypes than age-matched men, an advantage that narrows after menopause with the loss of estrogen-mediated vascular protection [36,37]. Obesity, particularly visceral adiposity, has been associated with carotid plaque progression and instability, potentially through adverse metabolic and inflammatory pathways [38,39]. Hypertension promotes carotid plaque development through increased mechanical wall stress and is associated with vulnerable plaque features like intraplaque hemorrhage and lipid-rich necrotic cores [40,41]. According to the 2023 ESC Guidelines for the Management of Cardiovascular Disease in Patients with Diabetes and the 2019 ESC/EAS Guidelines for the Management of Dyslipidemias, systemic comorbidities such as diabetes and dyslipidemia are determinants of atherosclerotic disease and plaque characteristics [42,43]. Metabolic syndrome, reflecting the clustering of hypertension, dysglycemia, obesity, dyslipidemia, and smoking, is associated with carotid plaque development and instability, with risk increasing progressively as the metabolic components rise, indicating that these factors frequently act together rather than in isolation [20,44,45,46]. Metabolic dysfunction-associated steatotic liver disease (MASLD) and hepatic fibrosis share several cardiometabolic and inflammatory pathways with atherosclerosis and have been associated with carotid plaque burden and vulnerability [47,48,49,50,51,52]. Although hepatic steatosis, liver stiffness, and fibrosis indices were not assessed in the present study, their potential influence warrants consideration, and incorporating these measures into future research may provide a more comprehensive understanding of the association between smoking and carotid plaque stiffness.

Several limitations should be acknowledged when interpreting these findings. As a single-center pilot study, this investigation provided preliminary evidence regarding the association between smoking and carotid plaque stiffness. Cigarette smoking status was classified as current smoker or non-smoker, without detailed information on smoking intensity, pack per day, or smoking duration. Potential confounders, including lipid levels and medication use, were not included in the analyses. In addition, small plaques and plaques affected by substantial acoustic shadowing were excluded because of the fixed minimum ROI diameter and the dependence of 2D-SWE on adequate B-mode image quality. Although standardized ROI placement at the thickest part of the plaque improved measurement consistency, it may not fully reflect spatial variations in stiffness within heterogeneous plaques. Plaque stiffness was assessed irrespective of its precise anatomical location within the carotid artery, and plaque composition was not confirmed histologically. Future multicenter longitudinal studies should consider larger samples, detailed assessment of tobacco exposure, relevant confounding variables, multiple-region or whole-plaque stiffness measurements, and histological validation when clinically feasible.

## 5. Conclusions

Carotid plaques in cigarette smokers demonstrated significantly greater stiffness than those in non-smokers, as indicated by higher ultrasound SWE-derived shear-wave velocity. These findings suggest that smoking is associated with alterations in the biomechanical properties of carotid atherosclerotic plaques and support SWE as a potential quantitative adjunct to conventional ultrasound assessment. However, higher plaque stiffness should not be interpreted as a direct marker of either plaque stability or vulnerability. Large prospective studies incorporating detailed smoking exposure, relevant cardiovascular confounders, histological assessment of plaque composition, and clinical outcomes are required.

## Figures and Tables

**Table 1:** Summary of patient characteristics, plaque distribution, and stiffness through shear-wave velocity.

	Study Population (n = 93 Patients)
*Patient characteristics*	
Age, mean ± SD [years]	68.4 ± 10.1
Gender, no [%]	
Men	73 [78.5]
Women	20 [21.5]
Hypertension, no [%]	
Yes	70 [75.3]
No	23 [24.7]
Diabetes, no [%]	
Yes	62 [66.7]
No	31 [33.3]
Smoking, no [%]	
Yes	45 [48.4]
No	48 [51.6]
*Plaque characteristics*	
Total number of plaques	132
Men, no. [%]	108 [81.8]
Women, no. [%]	24 [18.2]
Shear-wave velocity in m/s	
Mean ± SD	3.87 ± 1.40
Median [IQR]	3.79 [2.57]

Abbreviations: IQR, interquartile range; no., number; SD, standard deviation.

**Table 2:** Demographic and clinical characteristics, plaque distribution, and stiffness according to smoking status.

Characteristic	Smokers (n = 45)	Non-Smokers (n = 48)	*p*-Value
Patient characteristics			
Age, mean ± SD [years]	66.6 ± 10.1	70.1 ± 9.9	0.088
BMI, mean ± SD	24.2 ± 4.4	23.2 ± 4.8	0.255
Gender, no. [%]			
Men	36 [80.0]	37 [77.1]	0.929
Women	9 [20.0]	11 [22.9]	
Hypertension, no. [%]			
Yes	35 [77.8]	35 [72.9]	0.762
No	10 [22.2]	13 [27.1]	
Diabetes mellitus, no. [%]			
Yes	33 [73.3]	29 [60.4]	0.271
No	12 [26.7]	19 [39.6]	
Number of plaques per patient, no. [%]			
One plaque	33 [73.3]	35 [72.9]	—
More than one plaque	12 [26.7]	13 [27.1]	
Plaque distribution			
Total plaques, no.	65	67	—
Plaques in men, no. [%]	55 [84.6]	53 [79.1]	
Plaques in women, no. [%]	10 [15.4]	14 [20.9]	
SWE measurements			
Total SWE acquisitions, no.	195	201	—
Shear-wave velocity, median [IQR] [m/s]	4.17 [1.94]	2.91 [1.81]	<0.001

Abbreviations: IQR, interquartile range; no., number; SD, standard deviation; SWE, shear-wave elastography.

## Data Availability

The data presented in this study are available on request from the corresponding author due to ethical restrictions.
